# Frequencies of decision making and monitoring in adaptive resource management

**DOI:** 10.1371/journal.pone.0182934

**Published:** 2017-08-11

**Authors:** Byron K. Williams, Fred A. Johnson

**Affiliations:** 1 The Wildlife Society, Bethesda, Maryland, United States of America; 2 Southeast Ecological Science Center, U.S. Geological Survey, Gainesville, Florida, United States of America; US Army Engineer Research and Development Center, UNITED STATES

## Abstract

Adaptive management involves learning-oriented decision making in the presence of uncertainty about the responses of a resource system to management. It is implemented through an iterative sequence of decision making, monitoring and assessment of system responses, and incorporating what is learned into future decision making. Decision making at each point is informed by a value or objective function, for example total harvest anticipated over some time frame. The value function expresses the value associated with decisions, and it is influenced by system status as updated through monitoring. Often, decision making follows shortly after a monitoring event. However, it is certainly possible for the cadence of decision making to differ from that of monitoring. In this paper we consider different combinations of annual and biennial decision making, along with annual and biennial monitoring. With biennial decision making decisions are changed only every other year; with biennial monitoring field data are collected only every other year. Different cadences of decision making combine with annual and biennial monitoring to define 4 scenarios. Under each scenario we describe optimal valuations for active and passive adaptive decision making. We highlight patterns in valuation among scenarios, depending on the occurrence of monitoring and decision making events. Differences between years are tied to the fact that every other year a new decision can be made no matter what the scenario, and state information is available to inform that decision. In the subsequent year, however, in 3 of the 4 scenarios either a decision is repeated or monitoring does not occur (or both). There are substantive differences in optimal values among the scenarios, as well as the optimal policies producing those values. Especially noteworthy is the influence of monitoring cadence on valuation in some years. We highlight patterns in policy and valuation among the scenarios, and discuss management implications and extensions.

## Introduction

A well-known approach to learning-oriented decision making in natural resources is adaptive management, in which learning occurs through recursive management and what is learned at each time is used to guide future management actions (Williams and Brown [1–2]). Adaptive decision making is based on the recognition that resource systems are only partially understood, and there is value in tracking resource conditions and applying what is learned as the resources are being managed (Williams [3]). In the ongoing process of learning and adaptation, adjustments to decision making occur as understanding improves, with the ultimate goal of improved management (Walters [4]).

Adaptive decision making is by its nature flexible, and therefore is applicable to a wide variety of resource problems (Williams and Brown [5]). In some instances its focus is on the improvement of understanding about the role of management in influencing resource dynamics (Linkov et al. [6], Runge et al. [7]). In others it is on the social and institutional framework supporting iterative decision making (Susskind et al. [8], Convertino et al. [9]). In yet others, it is on the “architecture” of structured decision making, with the elicitation of values, objectives, decision alternatives etc. (Johnson et al. [10], Linkov et al. [11]). Even if one is primarily concerned about uncertainty and the improvement of technical understanding, the range of applicability is extremely broad. An important challenge for an adaptive framework is to cover a large number of decision problems, yet be flexible enough that it can be tailored to the details of any particular problem.

**Fig 1:**
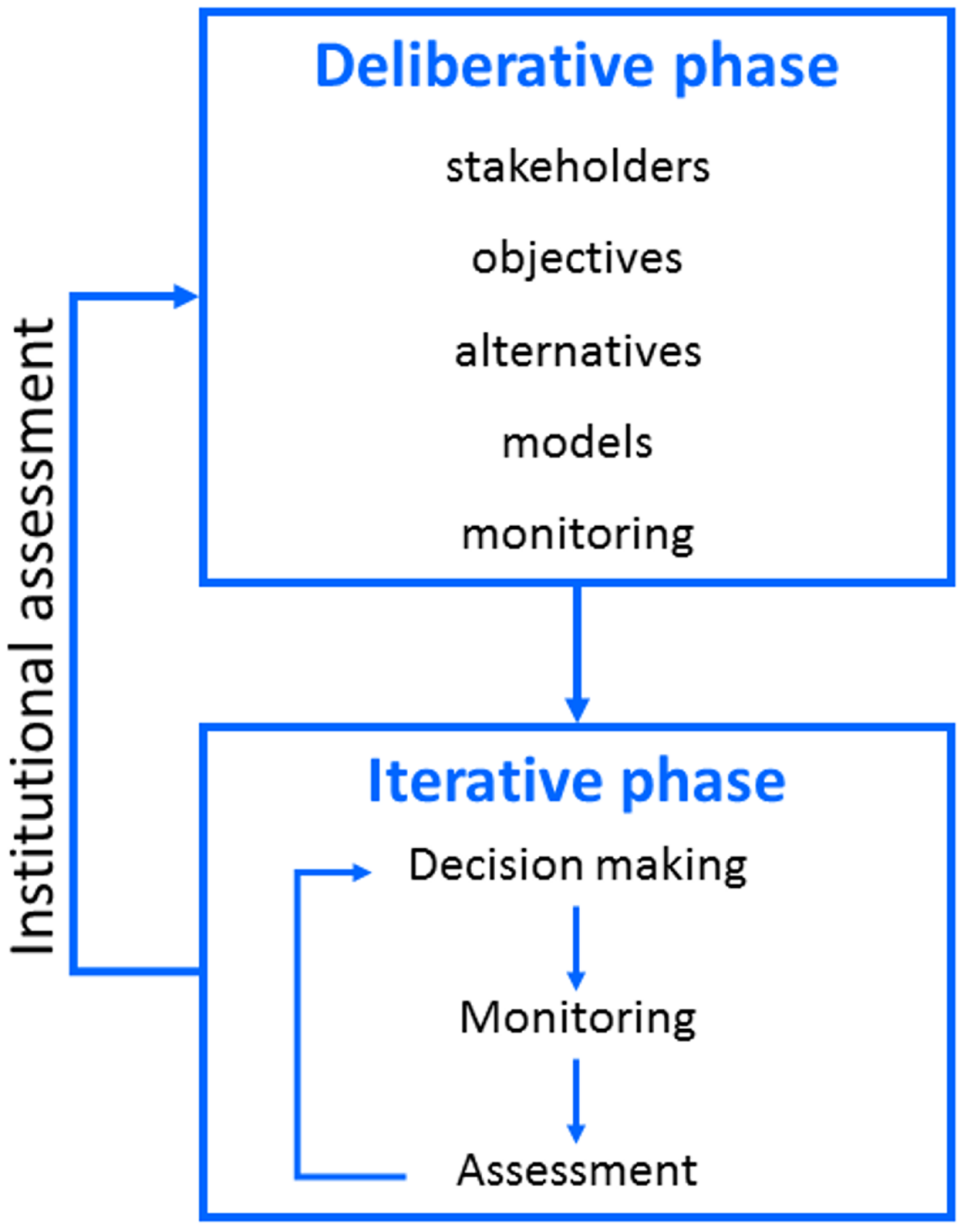
Learning in adaptive management. Technical learning involves an iterative sequence of decision making, monitoring, assessment, and feedback of what is learned into decision making. Institutional learning involves periodic reconsideration of the components in decision making (Williams and Brown [5]).

Here we take a formal, decision-theoretic approach to adaptive management (Johnson and Williams [12]), rather than more ad hoc approaches that sometimes are described in the literature (e.g., Schhreiber et al. [13]). In particular, we focus on an iterative sequencing of (*i*) decision making and taking actions, (*ii*) followed by monitoring of system responses, (*iii*) followed by assessment of data, (*iv*) followed by incorporating what is learned into future decision making (Fig 1). System state is typically monitored at fixed intervals, often annually, in order to inform decisions that occur with the same frequency (Hauser et al. [14]).

Alternatives to the coincidence of decision making and monitoring involve different cadences for the 2 activities. For example, the setting of migratory bird hunting regulations often involves annual monitoring and decision-making, but a different sequence was adopted for pink-footed geese (*Anser brachyrhynchus*) in Europe (Johnson and Madsen [15]). In the latter case, administrative burden is reduced by fixing harvest quotas for three years, while population monitoring occurs annually. Thus, while learning accrues annually, decisions are based on system state only every fourth year. In the United States, the regulations setting process for duck harvest has recently been modified so that current system state is not known (monitored) at the time a decision must be made (Johnson et al. [16]). Decisions must therefore be conditioned on the previous system state and regulatory action.

The issue of timing in decisions and monitoring has arisen in other decision processes as well. An example is the adaptive management program adopted by the Atlantic States Marine Fishery Commission for the establishment of horseshoe crab harvest in Delaware Bay quotas (Smith et al. [17]). State variables relevant to harvest decisions include not only the abundance of the harvested species, but also the abundance of migratory shorebirds (red knots [*Calidris canutus*]) that depend on horseshoe crab eggs as a food source at key migration stopover sites in Delaware Bay. Harvest quotas for the fishing season of June-December, year *t*+1 are established in the fall (e.g., November) of year *t*. The decisions are informed by estimates of system state variables obtained in May of year *t* (red knots) and October-November of year *t*-1 (horseshoe crabs [*Limulus polyphemus*]). Debate has ensued about the feasibility of pushing the harvest decision forward (e.g., January, year *t*+1) in order to make use of the previous fall’s crab survey data.

More generally, disconnecting the sequencing of decision making and monitoring is potentially advantageous, in that cost-savings often can be obtained by reducing the frequency of monitoring, or alternatively reducing the frequency of analysis and decision making. Of course, an important question concerns the effect of such asynchrony, in particular as it relates to the value produced by decisions made when there is restricted decision making or an absence of monitoring information. The issue can be framed in terms of the value produced with management policy that is informed by monitoring. One approach is to identify a value function, for example the expected accumulated harvest of a biological population, which expresses the value associated with decision making given the status of the resource being managed and some measure of understanding of it. If the resource is managed optimally based on current information about it, the question at issue is whether and to what degree the value produced through decision making is compromised by an asynchrony between decision making and monitoring.

Our objective here is to provide a framework by which to consider the question of asynchrony in monitoring and decision making, by describing and assessing valuation forms for 4 simple and plausible scenarios. These involve annual and biennial decision making in combination with annual and biennial monitoring. With biennial decision making, decisions are changed only every other year; with biennial monitoring, field data are collected only every other year. We acknowledge that other cadences are possible and could be considered. But we believe that the 4 scenarios developed here serve to highlight relevant patterns.

We first summarize the technical framework for adaptive decision making, and then describe value functions for each of 4 scenarios. For each scenario we describe optimal valuations and policies under both active and passive adaptive management.

## Decision making under structural uncertainty

A formal expression for adaptive management in the presence of structural uncertainty can be given in terms of a resource system that changes through time in response to iterative decision making, with models describing periodic change in resource status. The parameters and elements needed to characterize iterative decision making under uncertainty include:

*t*—time index for a range of times constituting the time frame. The index is assumed here to take positive integer values, starting at some time *t*_0_ and ending at time *T* which may be infinite. In what follows we also use *τ* as a time index, to represent forward aggregations of values conditional on some starting time *t*, as in $\sum_{\tau =t}^{T}a_{\tau }$.
*x*_*t*_—system state (size, density, spatial coverage, etc). Because the system is assumed to change through time its state is time-specific. It is assumed for now that system state is fully observable. We discuss the implications of partial observability below. In what follows we will need to consider the summation of values *f*(*x*_*t*_) across all system states for a given time *t*, which we abbreviate with the notation $\sum_{x_{t}}f(x_{t})$.
*k*—model index for *k* = 1,…,*K* models representing different hypotheses about system dynamics.
*q*_*t*_—vector (*q*_*t*_(1),*q*_*t*_(2),…,*q*_*t*_(*K*)) of probabilities, with *q*_*t*_(*K*) the probability that model *k* best represents the system at time *t*. The vector *q*_*t*_ is referred to as the model state, and it evolves through time as information accumulates via monitoring.
*a*_*t*_—action taken as a result of decision making. Because they are taken through time, actions are time-indexed.
*A*_*t*_—policy that specifies a particular action for each system state and model state at each time starting at time *t* in the time frame. *A*_0_ specifies actions over the full time frame {*t*_0_,…,*T*}, and *A*_*t*_ identifies the actions over a subset {*t*,…,*T*} of the time frame, starting at *t* ≥ *t*_0_.

### System dynamics

Here we assume that transitions among system states at any point in time are influenced by the current state but not previous states, and by the action taken at that time. That is, state transitions can be described as Markovian (Puterman [18], Williams et al. [19]). If *x*_*t*_ and *a*_*t*_ are the state and action at a particular time *t* and *x*_*t*+1_ is the state at the next time, then the probability of transition from *x*_*t*_ to *x*_*t*+1_ is *P*(*x*_*t*+1_ | *x*_*t*_,*a*_*t*_).

Structural uncertainty reflects an incomplete understanding of system dynamics, i.e., the transition probabilities in *P*(*x*_*t*+1_ | *x*_*t*_,*a*_*t*_) are uncertain (Williams [20], Williams and Brown [2]). Different Markovian models *P*_*k*_(*x*_*t*+1_ | *x*_*t*_,*a*_*t*_) along with an evolving model state can be used to account for structural uncertainty. Model-specific transition probabilities can be averaged based on *q*_*t*_, to produce

$$\bar{P}(x_{t+1}|x_{t},a_{t},q_{t})=\sum_{k}q_{t}(k)P_{k}(x_{t+1}|x_{t},a_{t}).$$

### Decision making

In the presence of structural uncertainty, policy is a function of the state of the system at time *t* and our understanding of system dynamics (and associated uncertainty) at time *t*, such that *A*(*x*_*t*_,*q*_*t*_) = *a*_*t*_. Policy *A*_*t*_ over a time frame {*t*,…,*T*} can be described sequentially by actions for each system and model state at time *t*, followed thereafter by the remainder *A*_*t*+1_ of the policy over {*t* + 1,…,*T*}:

$$A_{t}=\{A(x_{t},{\underline{q}}_{t}),A_{t+1}\}=\{a_{t},A_{t+1}\}.$$

In what follows it will be useful to consider decision making over 2 time steps, in which actions for 2 time steps are jointly determined. This situation is denoted by *A*_*t*_ = {*a*_*t*_,*a*_*t*+1_,*A*_*t*+2_}.

### Propagating uncertainty

Just as the system state evolves through time in response to management actions, so too does the model state (Williams and Johnson [21]). The dynamics of the model state are driven by the information produced through time with ongoing management, in the spirit of adaptive management (Nichols and Williams [22]). With iterative management, decision making influences an evolving system state *x*_*t*_, with transitions that are recognized through ongoing monitoring in turn influencing the level of uncertainty. Bayes’ theorem (Lee [23]) is used for updating uncertainty, based on system state transitions from *x*_*t*_ to *x*_*t*+1_:

$$\begin{array}{ll} q_{t+1}(k) & =\frac{q_{t}(k)P_{k}(x_{t+1}|x_{t},a_{t})}{\sum_{k}q_{t}(k)P_{k}(x_{t+1}|x_{t},a_{t})}\\ & =\frac{q_{t}(k)P_{k}(x_{t+1}|x_{t},a_{t})}{\bar{P}(x_{t+1}|x_{t},a_{t},{\underline{q}}_{t})}. \end{array}$$ (1)

Bayes’ theorem can also be used to determine the propagation of uncertainty across 2 time steps, as

$$q_{t+2}(k)=\frac{q_{t}(k)\sum_{x_{t+1}}P_{k}(x_{t+1}|x_{t},a_{t})P_{k}(x_{t+2}|x_{t+1},a_{t+1})}{\sum_{k}q_{t}(k)\sum_{x_{t+1}}P_{k}(x_{t+1}|x_{t},a_{t})P_{k}(x_{t+2}|x_{t+1},a_{t+1})},$$

which can be rewritten as

$$q_{t+2}(k)=\frac{q_{t}(k)\sum_{t+1}P_{k}(x_{t+1}\left|x_{t},a_{t})P_{k}(x_{t+2}\right|x_{t+1},a_{t+1})}{\sum_{x_{t+1}}\bar{P}(x_{t+1}\left|x_{t},a_{t},{\underline{q}}_{t})\bar{P}(x_{t+2}\right|x_{t+1},a_{t+1},{\underline{q}}_{t+1})}.$$ (2)

## Optimal decision making

Smart decision making requires an objective or value function to guide decisions and evaluate progress toward their achievement. Typically, valuation for adaptive management is based on the accrual of returns *R*(*x*_*t*_,*a*_*t*_) through time, with each return incorporating costs and benefits corresponding to action *a*_*t*_ when the system is in state *x*_*t*_ (Williams et al. [19]). A value function *V*(*A*_*t*_ | *x*_*t*_,*q*_*t*_) expresses the aggregation of returns associated with policy *A*_*t*_, given system state *x*_*t*_ and model state *q*_*t*_:

$$V(A_{t}|x_{t},{\underline{q}}_{t})=E\left[\sum_{\tau =t}^{T}R(x_{\tau },a_{\tau })|x_{t},\;{\underline{q}}_{t}\right],$$ (3)

where the expectation accounts for stochastic transitions among states through time as well as the structural uncertainty represented by multiple Markovian models *P*_*k*_(*x*_*t*+1_ | *x*_*t*_,*a*_*t*_) and their evolving probabilities *q*_*t*_(*k*). *V*(*A*_*t*_ | *x*_*t*_,*q*_*t*_) serves as an objective or value function by which to compare and contrast the effectiveness of different management strategies.

Two important variations of adaptive decision making are active and passive adaptive management. Active adaptive management incorporates the potential for learning directly into the process of decision making (Williams [24]). Thus, optimal active adaptive management accounts for system state and structural uncertainty at each decision point, and it also accounts explicitly for learning in the choice of strategy:

$$\begin{array}{ll} V\left[x_{t},{\underline{q}}_{t}\right] & =\underset{A_{t}}{\text{max}}V\left(A_{t}|x_{t},{\underline{q}}_{t}\right)\\ & =\underset{\left\{a_{t},A_{t+1}\right\}}{\text{max}}\left\{R(x_{t},a_{t})+\lambda \sum_{x_{t+1}}\bar{P}(x_{t+1}|x_{t},a_{t},{\underline{q}}_{t})V\left(A_{t+1}|x_{t+1},{\underline{q}}_{t+1}\right)\right\}\\ & =\underset{a_{t}}{\text{max}}\left\{R(x_{t},a_{t})+\lambda \sum_{x_{t+1}}\bar{P}(x_{t+1}|x_{t},a_{t},{\underline{q}}_{t})V\left[x_{t+1},{\underline{q}}_{t+1}\right]\right\}, \end{array}$$ (4)

where λ is a discount factor and the updated model state *q*_*t*+1_ in *V*[*x*_*t*+1_,*q*_*t*+1_] indicates the use of learning in identification of strategy. That is, the consequences of learning are anticipated in the decision making process itself. Active adaptive management via Eq (4) produces the optimal value of the function in Eq (3), i.e., maximum valuation in the face of structural uncertainty.

Active adaptive management can also be expressed in terms of 2 successive time periods by

$$\begin{array}{l} V\left[x_{t},{\underline{q}}_{t}\right]=\underset{\left\{a_{t},a_{t+1}\right\}}{\text{max}}\{R(x_{t},a_{t})+\lambda \sum_{x_{t+1}}\bar{P}(x_{t+1}|x_{t},a_{t},{\underline{q}}_{t})\\ \;\times \left[R(x_{t+1},a_{t+1})+\lambda^{2}\sum_{x_{t+2}}\bar{P}(x_{t+2}|x_{t+1},a_{t+1},{\underline{q}}_{t+1})V\left[x_{t+2},{\underline{q}}_{t+2}\right]\right]\}. \end{array}$$ (5)

where the term in brackets in Eq (5) is simply another expression for *V*[*x*_*t*+1_,*q*_*t*+1_]. The 2-step form for optimization in Eq (5) will prove to be especially useful in what follows for describing valuations of scenarios involving biennial patterns in decision making and monitoring.

With passive adaptive management, decision making is again based on system state and uncertainty at each decision point, but without explicitly accounting for learning in the choice of strategy (Williams [24]). The effect on valuation is seen by

$$\begin{array}{ll} V\left[x_{t},{\underline{q}}_{t}\right] & =\underset{A_{t}}{\text{max}}V\left(A_{t}|x_{t},{\underline{q}}_{t}\right)\\ & =\underset{\left\{a_{t},A_{t+1}\right\}}{\text{max}}\left\{R(x_{t},a_{t})+\lambda \sum_{x_{t+1}}\bar{P}(x_{t+1}|x_{t},a_{t},{\underline{q}}_{t})V\left(A_{t+1}|x_{t+1},{\underline{q}}_{t}\right)\right\}\\ & =\underset{a_{t}}{\text{max}}\left\{R(x_{t},a_{t})+\lambda \sum_{x_{t+1}}\bar{P}(x_{t+1}|x_{t},a_{t},{\underline{q}}_{t})V\left[x_{t+1},{\underline{q}}_{t}\right]\right\}, \end{array}$$ (6)

where the prior model state *q*_*t*_ in *V*[*x*_*t*+1_,*q*_*t*_] indicates the absence of learning in the identification of decisions. The corresponding form for 2-step passive adaptive optimization is

$$\begin{array}{l} V\left[x_{t},{\underline{q}}_{t}\right]=\underset{\left\{a_{t},a_{t+1}\right\}}{\text{max}}\{R(x_{t},a_{t})+\lambda \sum_{x_{t+1}}\bar{P}(x_{t+1}|x_{t},a_{t},{\underline{q}}_{t})\\ \;\times \left[R(x_{t+1},a_{t+1})+\lambda^{2}\sum_{x_{t+2}}\bar{P}(x_{t+2}|x_{t+1},a_{t+1},{\underline{q}}_{t})V\left[x_{t+2},{\underline{q}}_{t}\right]\right]\}. \end{array}$$ (7)

The only difference between active vs passive adaptive management as described above is the direct incorporation of learning into decision making, as indicated by an updated model state *q*_*t*+1_ in the valuation *V*[*x*_*t*+1_,*q*_*t*+1_] in Eq (4). In contrast, learning in passive adaptive management factors into future decision making only after the current decision is made. The absence of anticipated learning in guiding decisions is indicated by the use of current model state *q*_*t*_ in the value term *V*[*x*_*t*+1_,*q*_*t*_] in Eq (6). We note that our description of passive adaptive management extends beyond many descriptions in the literature, where passive adaptive management is held to involve actions based on the best available model, followed by post-decision monitoring to revise or replace the model (Walters and Hilborn [25], Schreiber et al. [13], Williams [24]).

While the value *V*[*x*_*t*_,*q*_*t*_] produced by passive adaptive management is necessarily less than that of active adaptive management, the passive form has the advantage of being computationally tractable for relatively large problems, specifically because only the current model state must be considered. In practice, policies and values may vary little between the active and passive forms (Johnson et al. [26], Hauser and Possingham [27]).

## Valuation under different cadences of decision making and monitoring

The learning-based approach described above involves iterative decision making through time, utilizing monitoring information that is collected at each decision point. However, the selection of decisions need not coincide with the monitoring of system transitions. In what follows we consider annual and biennial decision making along with annual and biennial monitoring, where biennial decision making involves changes in decisions only every other year and biennial monitoring means the collection of field data every other year. The options for decision making combine with those for monitoring to define 4 scenarios. Here we discuss optimal valuations for each scenario, and compare/contrast the valuations among scenarios. We acknowledge that variations in timing beyond the biennial cadences considered here are possible, and we highlight other examples in the discussion below.

Given the scenarios defined by annual and biennial cadences, every 2 years a new decision can be made and state information is available to inform that decision. In the subsequent year after a new decision, however, in 3 of the 4 scenarios either the decision is repeated or monitoring does not occur (or both). The difference among scenarios becomes clear by focusing on the arguments of the value function *V*(*A*_*t*_ | *x*_*t*_,*q*_*t*_).

- Scenario 1: Annual decision making and annual monitoring
  - Every year (*x*_*t*_,*q*_*t*_) is known because of annual monitoring
  - A new action can be taken every year
- Scenario 2: Annual decision making and biennial monitoring
  - Every other year (*x*_*t*_,*q*_*t*_) is not known because of the lack of monitoring
  - A new action can be taken every year
- Scenario 3: Biennial decision making and annual monitoring:
  - Every year (*x*_*t*_,*q*_*t*_) is known because of annual monitoring
  - The same action is taken in successive years
- Scenario 4: Biennial decision making and biennial monitoring
  - Every other year (*x*_*t*_,*q*_*t*_) is not known because of the lack of monitoring
  - The same action is taken in successive years

The differences among scenarios are accentuated in non-monitoring years. In this situation scenarios 1 and 2 produce different valuations, because the state is seen via monitoring under scenario 1 but not under scenario 2. Scenarios 3 and 4 also produce different valuations, for the same reason: the state is seen via monitoring under scenario 3 but not under scenario 4. Finally, the valuations for the scenarios 1 and 2 differ from those for scenarios 3 and 4, because actions in successive years are repeated in scenarios 3 and 4.

In the next sections we assume active adaptive decision making in the development of valuation forms. We then describe valuation under passive adaptive management. In both cases we use *V*(*A*_*t*_ | *x*_*t*_,*q*_*t*_) as in Eq (3) to represent the aggregate value associated with policy *A*_*t*_ given the combination (*x*_*t*_,*q*_*t*_) of system and model states, and use *V*[*x*_*t*_,*q*_*t*_] as in Eqs (5) and (7) to represent the optimal valuation obtained by maximizing *V*(*A*_*t*_ | *x*_*t*_,*q*_*t*_) over all available policies.

### Scenario 1: Valuation under annual decision making and monitoring

**Fig 2:**
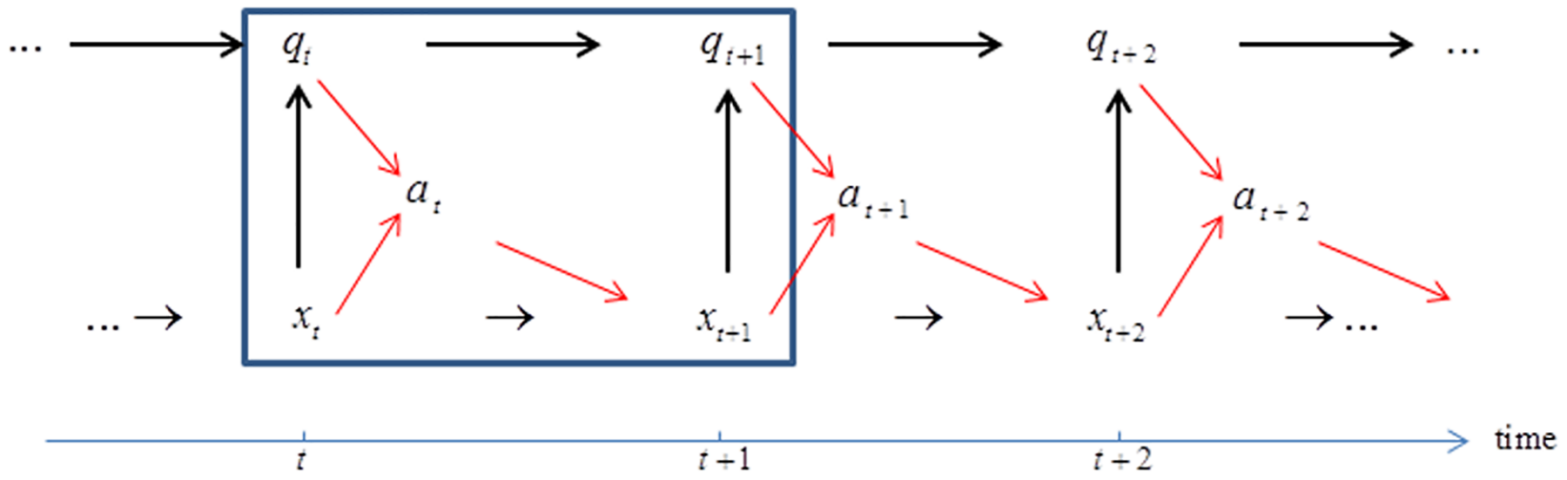
Scenario 1: Annual decision making and annual monitoring. Action *a*_*t*_ is selected based on system state *x*_*t*_ and model state *q*_*t*_. Realized system state *x*_*t*+1_ is identified through monitoring in year *t*+1. Model state *q*_*t*_ is updated to *q*_*t*+1_ with by Bayes’ theorem. This sequence, with actions based on current system and model state, is repeated over the remainder of the time frame.

Here we describe the standard scenario for dynamic optimization (Williams and Johnson [28], Bertsekas [29]), in which decisions can be changed every year and observations about resource status are available to identify optimal actions and values (Fig 2). Thus, in any year *t* a new action *a*_*t*_ can be selected based the system state *x*_*t*_ and model state *q*_*t*_. Immediately before the next decision point in year *t*+1 the system state *x*_*t*+1_ is identified through monitoring, and model-specific probabilities *P*_*k*_(*x*_*t*+1_ | *x*_*t*_,*a*_*t*_) of transition from system state *x*_*t*_ to *x*_*t*+1_ are identified. These transition probabilities are combined with the model state *q*_*t*_ to produce an updated model state *q*_*t*+1_ by Bayes’ theorem (Eq 1). The updated system and model states are then available to inform the selection of an action *a*_*t*+1_ at year *t*+1.

The determination of optimal values and actions is facilitated with recursion approaches (Puterman [18]). In a given year *t* the value function can be expressed recursively as

$$V\left(A_{t}|x_{t},{\underline{q}}_{t}\right)=R(x_{t},a_{t})+\lambda \sum_{x_{t+1}}\bar{P}(x_{t+1}|x_{t},a_{t},{\underline{q}}_{t})V\left(A_{t+1}|x_{t+1},{\underline{q}}_{t+1}\right),$$ (8)

and maximization

$$V\left[x_{t},{\underline{q}}_{t}\right]=\underset{a_{t}}{\text{max}}\left\{R(x_{t},a_{t})+\lambda \sum_{x_{t+1}}\bar{P}(x_{t+1}|x_{t},a_{t},{\underline{q}}_{t})V\left[x_{t+1},{\underline{q}}_{t+1}\right]\right\}$$ (9)

over *A*_*t*_ = {*a*_*t*_,*A*_*t*+1_} produces $a_{t}^{*}$ and *V*[*x*_*t*_,*q*_*t*_] for each (*x*_*t*_,*q*_*t*_).

Because new actions can be taken every year and system status is always observed, valuation in successive years *t* and *t*+1 have the same form, with the value function for *t*+1 replicating Eq (8) simply by incrementing the time index by 1:

$$V\left(A_{t+1}|x_{t+1},{\underline{q}}_{t+1}\right)=R(x_{t+1},a_{t+1})+\lambda \sum_{x_{t+2}}\bar{P}(x_{t+2}|x_{t+1},a_{t+1},{\underline{q}}_{t+1})V\left(A_{t+2}|x_{t+2},{\underline{q}}_{t+2}\right).$$ (10)

An algorithm for determining optimal values and policies with scenario 1 is discussed in the Appendix.

### Scenario 2: Valuation under annual decision making and biennial monitoring

**Fig 3:**
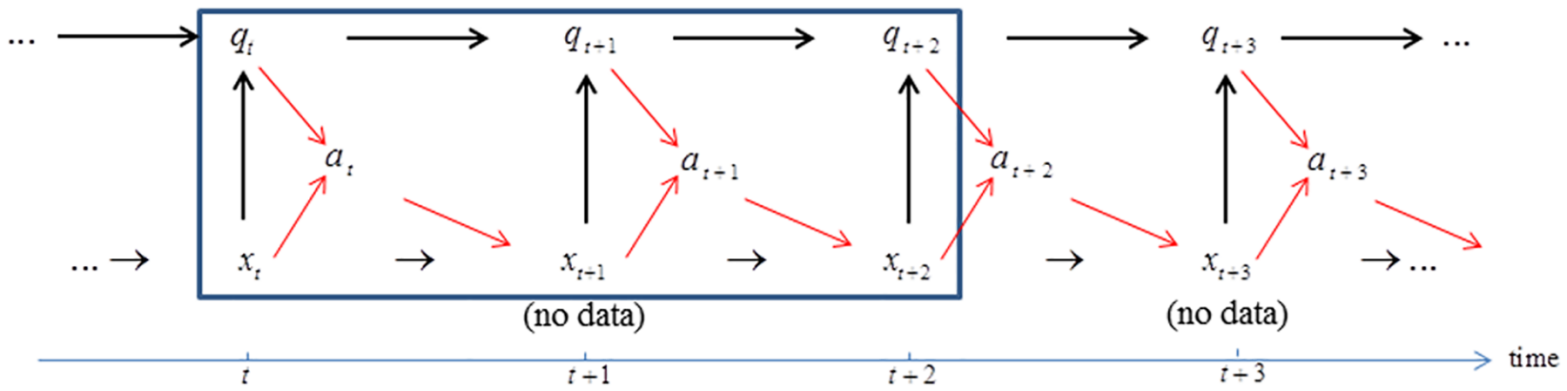
Scenario 2: Annual decision making and biennial monitoring. Actions *a*_*t*_ and *a*_*t*+1_ are jointly selected based on system state *x*_*t*_ and model state *q*_*t*_. Realized system state *x*_*t*+2_ is identified through monitoring in year *t*+2. Model state *q*_*t*_ is updated to *q*_*t*+2_ by Bayes’ theorem. This sequence, with actions *a*_*t*_ and *a*_*t*+1_ jointly chosen for successive years, is repeated over the remainder of the time frame.

In this scenario decisions can be changed each year as in the standard scenario 1, but the monitoring by which system and model states are recognized occurs only every other year. If system state is observed in a given year *t*, not observed in the subsequent year *t*+1, and observed again in year *t*+2, a 2-step transition process is required (Fig 3). With state information *x*_*t*_ and *x*_*t*+2_ in years *t* and *t*+2, model-specific probabilities of transition from state *x*_*t*_ to *x*_*t*+2_ can be determined. These transition probabilities can be combined with model state *q*_*t*_ to determine model state *q*_*t*+2_ by Bayes’ theorem (Eq (2).

A 2-step value function

$$V(A_{t}|x_{t},{\underline{q}}_{t})=R(x_{t},a_{t})+\lambda \sum_{x_{t+1}}\bar{P}(x_{t+1}|x_{t},a_{t},{\underline{q}}_{t})V(A_{t+1}|x_{t+1},{\underline{q}}_{t+1}),$$ (11)

with

$$V(A_{t+1}|x_{t+1},{\underline{q}}_{t+1})=R(x_{t+1},a_{t+1})+\lambda \sum_{x_{t+2}}\bar{P}(x_{t+2}|x_{t+1},a_{t+1},{\underline{q}}_{t+1})V(A_{t+2}|x_{t+2},{\underline{q}}_{t+2})$$ (12)

allows optimal actions for year *t* and *t*+1 to be jointly identified. Maximizing *V*(*A*_*t*_ | *x*_*t*_,*q*_*t*_) over *A*_*t*_ = {*a*_*t*_,*a*_*t*+1_,*A*_*t*+2_} (Eq (5)) produces $a_{t}^{*}$,$a_{t+1}^{*}$,$A_{t+2}^{*}$ and *V*[*x*_*t*_,*q*_*t*_] for each combination (*x*_*t*_,*q*_*t*_).

Determining value in the subsequent year *t*+1 requires a somewhat different treatment. Because there is no monitoring in year *t*+1, the states *x*_*t*+1_ and *q*_*t*+1_ in Eq (12) are unknown. However, they are related stochastically to *x*_*t*_ and *q*_*t*_, which are known through monitoring. Averaging over the transition probabilities produces a valuation for year *t*+1 of

$$\bar{V}(A_{t+1}|x_{t},{\underline{q}}_{t},a_{t})=\sum_{x_{t+1}}\bar{P}(x_{t+1}|x_{t},a_{t},{\underline{q}}_{t})V(A_{t+1}|x_{t+1},{\underline{q}}_{t+1}),$$ (13)

and using $a_{t}^{*}$,$a_{t+1}^{*}$ and $A_{t+2}^{*}$ from the 2-step optimization in Eq (11) produces the optimal valuation

$$\bar{V}\left[x_{t},{\underline{q}}_{t},a_{t}^{*}\right]=\sum_{x_{t+1}}\bar{P}(x_{t+1}|x_{t},a_{t}^{*},{\underline{q}}_{t})V\left[x_{t+1},{\underline{q}}_{t+1}\right]$$ (14)

For year *t*+1. Note that the function in Eq (13) describing valuation for year *t*+1 has arguments that are indexed for the previous year *t*. The triple (*x*_*t*_,*q*_*t*_,*a*_*t*_) in $\bar{V}(A_{t+1}|x_{t},{\underline{q}}_{t},a_{t})$, inherited from $\bar{P}(x_{t+1}|x_{t},a_{t},{\underline{q}}_{t})$, is needed to anchor the transition from the previous year *t*, when system state *x*_*t*_ is known, to year *t*+1 when system state *x*_*t*+1_ is not known.

Computations of values and identification of policies for scenario 2 are discussed in the Appendix.

### Scenario 3: Valuation under biennial decision making and annual monitoring

**Fig 4:**
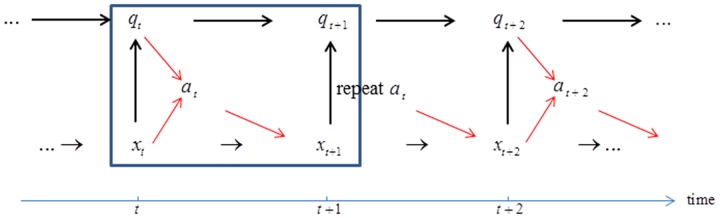
Scenario 3: Biennial decision making and annual monitoring. Action *a*_*t*_ is selected based on system state *x*_*t*_ and model state *q*_*t*_. Realized system state *x*_*t*+1_ is identified through monitoring in year *t*+1. Model state *q*_*t*_ is updated to *q*_*t*+1_ with Bayes’ theorem. Action *a*_*t*_ is repeated in year *t*+1. This sequence, with the same action taken in successive years, is repeated over the remainder of the time frame.

In this scenario monitoring occurs every year, as in the standard situation involving annual monitoring and decision making, but decisions can be changed only every other year. The sequencing of actions is as described above for scenario 1, except that every other year the action for the previous year is repeated (Fig 4). For a year *t* in which a new action can be taken, valuation that includes the repetition of actions in successive years is

$$V(A_{t}\prime |x_{t},{\underline{q}}_{t})=R(x_{t},a_{t})+\lambda \sum_{x_{t+1}}\bar{P}(x_{t+1}|x_{t},a_{t},{\underline{q}}_{t})V(A_{t+1}\prime |x_{t+1},{\underline{q}}_{t+1},a_{t}).$$ (15)

The conditioning argument *a*_*t*_ in *V*(*A*_*t*+1_′ | *x*_*t*+1_,*q*_*t*+1_,*a*_*t*_) is used here to emphasize that *a*_*t*+1_, the lead action in *A*_*t*+1_′ = {*a*_*t*+1_,*A*_*t*+2_′}, is predetermined to be *a*_*t*+1_ = *a*_*t*_ because of the biennial decision making. Maximizing over *A*_*t*_ = {*a*_*t*_,*a*_*t*_,*A*_*t*+2_} produces $a_{t}^{*}$, $a_{t+1}^{*}=a_{t}^{*}$, $A_{t+2}^{*}\prime$ and *V*′[*x*_*t*_,*q*_*t*_]. The “′” symbol in *A*_*t*_′ and *V*′[*x*_*t*_,*q*_*t*_] distinguishes strategies and valuations in scenario 3 from *V*(*A*_*t*_ | *x*_*t*_,*q*_*t*_) and *V*′[*x*_*t*_,*q*_*t*_] in scenario 1, where decisions can be changed annually. On inspection the only difference between the valuation *V*(*A*_*t*_′ | *x*_*t*_,*q*_*t*_) here and *V*(*A*_*t*_ | *x*_*t*_,*q*_*t*_) in Eq (8) for the standard scenario 1 is the replacement of *a*_*t*+1_ in Eq (15) with *a*_*t*_ in the computation of future returns. Of course, that seemingly marginal policy difference can have substantive consequences for valuation, depending on the Markovian structure of the problem.

Assuming that a new action can be taken in year *t* and is repeated in the subsequent year, the value function for year *t*+1 is

$$V(A_{t+1}\prime |x_{t+1},{\underline{q}}_{t+1},a_{t})=R(x_{t+1},a_{t})+\lambda \sum_{x_{t+2}}\bar{P}(x_{t+2}|x_{t+1},a_{t},{\underline{q}}_{t+1})V(A_{t+2}\prime |x_{t+2},{\underline{q}}_{t+2}).$$ (16)

Policy maximization for year *t*+1 then produces

$$\begin{array}{ll} V\prime \left[x_{t+1},{\underline{q}}_{t+1},a_{t}^{*}\right] & =\underset{A_{t+1}\prime }{\text{max}}V(A_{t+1}\prime |x_{t+1},{\underline{q}}_{t+1},a_{t}^{*})\\ & =R(x_{t+1},a_{t}^{*})+\lambda \sum_{x_{t+2}}\bar{P}(x_{t+2}|x_{t+1},a_{t}^{*},{\underline{q}}_{t+1})V\prime \left[x_{t+2},{\underline{q}}_{t+2}\right], \end{array}$$ (17)

where $a_{t}^{*}$ in $V\prime \left[x_{t+1},{\underline{q}}_{t+1},a_{t}^{*}\right]$ is identified by optimizing in value function in Eq (15). The action corresponding to the value $V\prime \left[x_{t+1},{\underline{q}}_{t+1},a_{t}^{*}\right]$ is of course $a_{t+1}^{*}=a_{t}^{*}$.

The determination of optimal values and policies with scenario 3 is discussed in the Appendix.

### Scenario 4: Valuation under biennial decision making and monitoring

**Fig 5:**
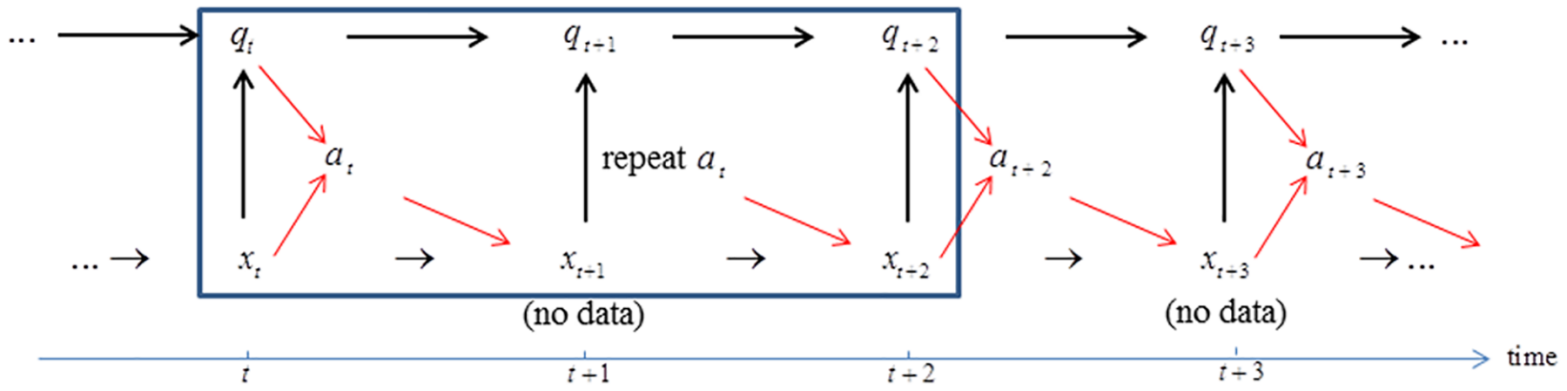
Scenario 4: Biennial decision making and biennial monitoring. Actions *a*_*t*_ and *a*_*t*+1_ = *a*_*t*_ are selected based on system state *x*_*t*_ and model state *q*_*t*_. Realized system state *x*_*t*+2_ is identified through monitoring in year *t*+2. Model state *q*_*t*_ is updated to *q*_*t*+2_ with Bayes’ Theorem. This sequence, with the same action chosen in successive years, is repeated over the remainder of the time frame.

Finally, decision making and monitoring can both be biennial, with decisions repeated and monitoring conducted only every other year (Fig 5). Valuation in a year *t* where a new action can be taken and monitoring occurs is given by

$$V(A_{t}\prime |x_{t},{\underline{q}}_{t})=R(a_{t},x_{t})+\lambda \sum_{x_{t+1}}\bar{P}(x_{t+1}|x_{t},a_{t},{\underline{q}}_{t})V(A_{t+1}\prime |x_{t+1},{\underline{q}}_{t+1},a_{t}),$$ (18)

With *a*_*t*_ in *V*(*A*_*t*+1_′ | *x*_*t*+1_,*q*_*t*+1_,*a*_*t*_) again used as a conditioning argument to emphasize that *a*_*t*+1_, the lead action in *A*_*t*+1_′ = {*a*_*t*+1_,*A*_*t*+2_′}, is predetermined to be *a*_*t*+1_ = *a*_*t*_ because of biennial decision making. Maximizing *V*(*A*_*t*_′ | *x*_*t*_,*q*_*t*_) in Eq (18) over *A*_*t*_′ = {*a*_*t*_,*a*_*t*_,*A*_*t*+2_′} produces $a_{t}^{*}$, $a_{t+1}^{*}=a_{t}^{*}$, $A_{t+2}^{*}\prime$ and *V*′[*x*_*t*_,*q*_*t*_]. This is the same form for scenario 3 with biennial decision making and annual monitoring.

However, scenario 4 differs from the other scenarios in the subsequent year *t*+1, because the conditioning states *x*_*t*+1_ and *q*_*t*+1_ are not known in the absence of monitoring. But their linkage to *x*_*t*_ and *q*_*t*_ can be used with *a*_*t*_ and *a*_*t*+1_ = *a*_*t*_ to produce the average value

$$\bar{V}(A_{t+1}\prime |x_{t},{\underline{q}}_{t},a_{t})=\sum_{x_{t+1}}\bar{P}(x_{t+1}|x_{t},a_{t},{\underline{q}}_{t})V(A_{t+1}\prime |x_{t+1},{\underline{q}}_{t+1},a_{t}),$$ (19)

with an optimal valuation of

$$\bar{V}\prime \left[x_{t},{\underline{q}}_{t},a_{t}^{*}\right]=\sum_{x_{t+1}}\bar{P}(x_{t+1}|x_{t},a_{t}^{*},{\underline{q}}_{t})V\prime \left[x_{t+1},{\underline{q}}_{t+1},a_{t}^{*}\right]$$ (20)

In year *t*+1. Eq (19) differs from the corresponding Eq (13) for scenario 2 with annual decision making and biennial monitoring, but only in that scenario 2 allows for different actions *a*_*t*_ and *a*_*t*+1_ in *A*_*t*_ = {*a*_*t*_,*a*_*t*+1_,*A*_*t*+2_}, whereas scenario 4 uses the same actions *a*_*t*_ and *a*_*t*+1_ = *a*_*t*_ in *A*_*t*_′ = {*a*_*t*_,*a*_*t*_,*A*_*t*+2_′}.

Computing forms for optimal values and policies with scenario 4 are discussed in the Appendix.

## Patterns in the optimal valuations

Several informative comparisons can be recognized among the scenarios, in terms of the actions to be optimized and the state information that is available when actions are to be selected.

### Comparisons of annual and biennial monitoring

For the scenarios considered here, in a year *t* when monitoring is conducted and new decisions are made the valuation of optimal policy is the same irrespective of monitoring frequency. That is, the same expression for optimal valuation obtains under both monitoring regimes. For annual decision making the optimal valuation for annual decision making is *V*[*x*_*t*_,*q*_*t*_] irrespective of the cadence of monitoring. For biennial decision making the optimal valuation is *V*′[*x*_*t*_,*q*_*t*_]. Basically, if one knows the system and model states when decisions are made, there is no additional value in collecting more information between decision points (but see below on the potential influence of partial observability). This pattern holds for annual as well as biennial decision making.

The situation is somewhat different for years when monitoring may not be conducted. Assume that monitoring occurs in year *t*, and may or may not in year *t*+1 depending on the scenario. With annual decision making and annual monitoring (scenario 1), in year *t*+1 one optimizes

$$V(A_{t+1}|x_{t+1},{\underline{q}}_{t+1})$$

as in Eq (10), whereas with biennial monitoring (scenario 2) one optimizes the average value

$$\bar{V}(A_{t+1}|x_{t},{\underline{q}}_{t},a_{t})=\sum_{x_{t+1}}\bar{P}(x_{t+1}|x_{t},a_{t},{\underline{q}}_{t})V(A_{t+1}|x_{t+1},{\underline{q}}_{t+1})$$

in Eq (13).

A similar pattern holds for biennial decision making. Under annual monitoring (scenario 3) one optimizes

$$V(A_{t+1}\prime |x_{t+1},{\underline{q}}_{t+1},a_{t})$$

in Eq (16), whereas under biennial monitoring (scenario 4) one optimizes the average value

$$\bar{V}(A_{t+1}\prime |x_{t},{\underline{q}}_{t},a_{t})=\sum_{x_{t+1}}\bar{P}(x_{t+1}|x_{t},a_{t},{\underline{q}}_{t})V(A_{t+1}\prime |x_{t+1},{\underline{q}}_{t+1},a_{t})$$

in Eq (19).

On reflection, these results make sense. The averaging with biennial monitoring is essentially a way to compensate for the lack of knowledge about system and model states at *t*+1. The effect of averaging clearly distinguishes the scenarios with biennial monitoring from those with annual monitoring, in their valuations as well as their policies.

### Comparisons of annual and biennial decision making

Even for years in which system state is observed, valuation varies with the cadence of decision making. As seen above, value is optimized for biennial decision making over *A*_*t*_′ = {*a*_*t*_,*a*_*t*_,*A*_*t*+2_′} rather than *A*_*t*_ = {*a*_*t*_,*a*_*t*+1_,*A*_*t*+2_} for annual decision making. The use of identical actions in successive years is definitive of biennial decision making. The same pattern holds for both annual as well as biennial monitoring.

It also holds for years *t*+1 in which system state is not necessarily observed. Of course, with biennial monitoring valuation involves the averaging of value functions in the absence of monitoring information.

## Passive adaptive management under different cadences

The value functions above are based on an active form of adaptive decision making, in which at any particular point in time the effect of learning is factored into future decision making (Eq (4)). An alternative to active adaptive management is passive adaptive management, in which learning influences future decision making only indirectly, after the current decision is made (Eq (6)).

Consider, for example, annual decision making and biennial monitoring (scenario 2) under passive adaptive management. The associated value function differs from that for active adaptive management only in the use of a stationary model state. In a year *t* when the system is observed the value function for passive adaptive management is

$$V(A_{t}|x_{t},{\underline{q}}_{t})=R(x_{t},a_{t})+\lambda \sum_{x_{t+1}}\bar{P}(x_{t+1}|x_{t},a_{t},{\underline{q}}_{t})V(A_{t+1}|x_{t+1},{\underline{q}}_{t}),$$ (21)

and in year *t*+1 when it is not the value function is

$$\bar{V}(A_{t+1}|x_{t},{\underline{q}}_{t},a_{t})=\sum_{x_{t+1}}\bar{P}(x_{t+1}|x_{t},a_{t},{\underline{q}}_{t})V(A_{t+1}|x_{t+1},{\underline{q}}_{t}).$$ (22)

These are the same forms as for active adaptive management (Eqs (11) and (13)), except for the use of a stationary model state in the transition probabilities and future values.

An analogous pattern can be seen for passive adaptive management under biennial decision making and annual monitoring (scenario 3). The value function for scenario 3 again differs from that for active adaptive management only in the model states used in the transition probabilities and future values. Thus, for a year when a new action can be selected the value function is

$$V(A_{t}\prime |x_{t},{\underline{q}}_{t})=R(a_{t},x_{t})+\lambda \sum_{x_{t+1}}\bar{P}(x_{t+1}|x_{t},a_{t},{\underline{q}}_{t})V(A_{t+1}\prime |x_{t+1},{\underline{q}}_{t})$$ (23)

where the current model state *q*_*t*_ is used in the transition probabilities and the future value *V*(*A*_*t*+2_′ | *x*_*t*+2_,*q*_*t*_). For a year in which the previous action is repeated the value function is

$$V(A_{t}\prime |x_{t},{\underline{q}}_{t},a_{t-1})=R(a_{t-1},x_{t})+\lambda \sum_{x_{t+1}}\bar{P}(x_{t+1}|x_{t},a_{t-1},{\underline{q}}_{t})V(A_{t+1}\prime |x_{t+1},{\underline{q}}_{t}).$$ (24)

These again are the same forms as for active adaptive management (Eqs (15) and (16)), except for the use of a stationary model state in the transition probabilities and future values.

In like manner, the value functions for scenarios 1 and 4 can be described on assumption that decision making is passive rather than active. In each of the 4 scenarios the passive adaptive management forms can be derived by simply restricting the model states in active adaptive management to be stationary. This reduces considerably the computational burden in identifying optimal policies and values.

## Discussion

As adaptive management continues to grow in its popularity and use in natural resources, there is a trend toward being more flexible in its implementation. But greater flexibility in turn creates new challenges in capturing an appropriate decision making “architecture” for individual problems. An important example concerns the frequencies of decision making and monitoring. In particular, an accounting is needed of the effects of different cadences on both strategy and valuation. Here we have described a technical framework that allows for assessment of differing combinations of annual and biennial decision making and monitoring.

In this paper we have highlighted substantive differences in value functions for varying cadences of decision making and monitoring, recognizing that the differences are less pronounced for years when a system is observed. Indeed, for a year *t* with observed status the cadence of monitoring does not affect valuation (or policy) for either annual or biennial decision making. On the other hand, the cadence of monitoring does affect valuation and policy for year *t*+1 in which the system is not observed.

These patterns provide insight into the value of the information that is added with more frequent monitoring (Yokota and Thompson [30], Williams and Johnson [28]). For example, in a monitoring year the valuations under less frequent and more frequent monitoring are identical, so no value is added by increasing the frequency of monitoring. But there is an added value in the subsequent year, as a result of the need to average the valuations across system states with less frequent monitoring. The gain in value with more frequent monitoring is the difference between a valuation informed by knowledge of system state (annual monitoring), versus an average valuation when system state is only known stochastically (biennial monitoring). The results of such an assessment can be useful to managers as a metric in determining whether to reduce annual to biennial monitoring, or to expand biennial to annual monitoring.

The assessment for annual and biennial cadences can be extended to include options for 3 or more years. Thus, one could consider the effect of decision making every 3 years rather than every year, or the effect of monitoring every 3 years rather than every year (Johnson and Madsen [15]). One way to assess such an extension would be to express returns in the value functions in terms of 3 time steps rather than 2, and compute the corresponding expectations. The practical effect would be to complicate the mathematical expressions for valuation, and likely would make more difficult the comparative interpretation of patterns.

Other variations in the cadence of monitoring and decision making are possible. In the above, biennial monitoring and decision making occur in the same years, which allows decisions to be informed by system and model states at those times. Another variation is for monitoring and decision making to occur in alternative years, for example with decision making in one year and monitoring to occur in the subsequent year. The overall effect of this cadence is to require averaging based on prior year status each time a new decision is made. Yet another variation involves decision making prior to monitoring each year, so that the monitoring results are not available to inform the selection of actions for that year (Johnson et al. [16]). Under these conditions, possible actions must again be conditioned on the previous system and model state and the action previously taken.

The lack of additional value in collecting information between decision points that is highlighted here depends on the assumption that the resource system is fully observable. An allowance for partial observability defines a partially observable Markov decision process (POMDP), in which system status is approximated by a time-specific probability distribution or “belief state” that is updated with monitoring data through time (Kaelbling et al. [31], Littman [32]). A natural accounting of both partial observability and structural uncertainty considers the updating of belief, whenever it occurs, as a factor in the updating of model state, so that a change is belief affects the propagation of model state and in so doing may influence policy and valuation. Under these circumstances monitoring between decision times can have an effect on decision making.

There is a very large technical literature on theory and applications of adaptive management in natural resources for fully observable systems, and a much smaller but growing literature of POMDP methods and applications in natural resources (e.g., Lane, D. [33], Chadés et al. [34], Haight and Polasky [35], Tomberlin [36], Chadés et al. [37], Fackler and Haight [38], Regan et al. [39], Nicol and Chadés [40]). However, there are very few expositions concerning natural resources that include both (Williams [20], Fackler and Pacifici [41], Chadés et al. [42]), even though structural uncertainty and partial observability are common in natural resources. The limited documentation is no doubt a result, at least partially, of the formidable difficulties of incorporating both factors in analytic and computational frameworks that are accessible to natural resources specialists (e.g., Jaulmes et al. [43], Williams [20], Bertsekas [29]). One rather ad hoc approach is to assume full observability, identify optimal policies and valuations as approximations to the broader problem that includes partial observability, and explore the sensitivity of the approximations to errors in state estimation.

Finally, a key determinant in the usefulness of comparative valuation with different cadences is the ability to actually compute values with the forms of the value functions discussed above. Software is currently available for active and passive adaptive management under annual decision making and monitoring (Lubow [44], Fackler [45]). It is straightforward to use this software for the case of biennial decision making and monitoring for passive adaptive management, by utilizing the 2-step transition probabilities. Further software development is necessary for the other scenarios, which involves a greater or lesser degree of difficulty and programming effort depending on the scenario.

## Appendix

In this appendix we outline computing algorithms and forms for the 4 scenarios. In each scenario the determination of optimal values and actions can be determined recursively.

### Scenario 1: Annual decision making and annual monitoring

In any given year *t* the value function can be expressed recursively as

$$V\left(A_{t}|x_{t},{\underline{q}}_{t}\right)=R(x_{t},a_{t})+\lambda \sum_{x_{t+1}}\bar{P}(x_{t+1}|x_{t},a_{t},{\underline{q}}_{t})V\left(A_{t+1}|x_{t+1},{\underline{q}}_{t+1}\right),$$

and maximization

$$V\left[x_{t},{\underline{q}}_{t}\right]=\underset{a_{t}}{\text{max}}\left\{R(x_{t},a_{t})+\lambda \sum_{x_{t+1}}\bar{P}(x_{t+1}|x_{t},a_{t},{\underline{q}}_{t})V\left[x_{t+1},{\underline{q}}_{t+1}\right]\right\}$$

over *A*_*t*_ = {*a*_*t*_,*A*_*t*+1_} produces $a_{t}^{*}$ and *V*[*x*_*t*_,*q*_*t*_] for each (*x*_*t*_,*q*_*t*_). This algorithm typically is applied sequentially throughout the time frame, starting at the terminal time *T* and stepping backward in single time steps (Williams et al. 2002, Bertsekas 2017).

### Scenario 2: Annual decision making and biennial monitoring

A recursive algorithm for identifying optimal valuation and strategy for scenario 2 involves a 2-step backward iteration to determine *V*[*x*_*t*_,*q*_*t*_] for a year *t* with monitoring, and with the results used to determine optimal valuation for year *t*+1. In the first step, *V*[*x*_*t*_,*q*_*t*_] is computed as above (Eq (9)), along with $a_{t}^{*}\;$ and $a_{t+1}^{*}$ for each combination (*x*_*t*_,*q*_*t*_). In the second step the optimal strategy $\left\{a_{t}^{*},a_{t+1}^{*},A_{t+2}^{*}\right\}$ is used to compute the average valuation $\bar{V}\left[x_{t},{\underline{q}}_{t},a_{t}^{*}\right]$ for year *t*+1 as shown in Eq (14).

### Scenario 3: Biennial decision making and annual monitoring

A recursive algorithm for identifying optimal valuation and strategy for scenario 3 again involves a 2-step iteration for any year *t* in which a new decision can be made, to determine *V*′[*x*_*t*_,*q*_*t*_] and $A_{t}^{*}\prime =\left\{a_{t}^{*},a_{t}^{*},A_{t+2}^{*}\prime \right\}$, and then use the results to determine $V\prime \left[x_{t+1},{\underline{q}}_{t+1},a_{t}^{*}\right]$ in year *t*+1. In the first step, *V*′[*x*_*t*_,*q*_*t*_] is computed for each combination (*x*_*t*_,*q*_*t*_) via the maximization of Eq (15), and the optimal action $a_{t}^{*}$ corresponding to (*x*_*t*_,*q*_*t*_) is identified. In the second step $a_{t}^{*}$ is used to compute $V\prime \left[x_{t+1},{\underline{q}}_{t+1},a_{t}^{*}\right]$ for year *t*+1 as in Eq (17), for each combination (*x*_*t*+1_,*q*_*t*+1_) in the triple $\left(x_{t+1},{\underline{q}}_{t+1},a_{t}^{*}\right)$.

### Scenario 4: Biennial decision making and biennial monitoring

A recursive algorithm for identifying optimal valuation and strategy for scenario 4 involves a 2-step backward iteration to determine *V*′[*x*_*t*_,*q*_*t*_] for each year *t* when monitoring occurs, and then using the results to determine optimal valuation for year *t*+1. In the first step, *V*′[*x*_*t*_,*q*_*t*_] is computed along with $a_{t}^{*}$ and $a_{t+1}^{*}=a_{t}^{*}$ for each combination (*x*_*t*_,*q*_*t*_), via the maximization of Eq (18). In the second step the optimal strategy $A_{t}^{*}\prime =\left\{a_{t}^{*},a_{t}^{*},A_{t+2}^{*}\prime \right\}$ is used to compute the average valuation for year *t*+1 with Eq (20).
